# A Review on Coordination Properties of Al(III) and Fe(III) toward Natural Antioxidant Molecules: Experimental and Theoretical Insights

**DOI:** 10.3390/molecules26092603

**Published:** 2021-04-29

**Authors:** Luana Malacaria, Giuseppina Anna Corrente, Amerigo Beneduci, Emilia Furia, Tiziana Marino, Gloria Mazzone

**Affiliations:** Department of Chemistry and Chemical Technologies, University of Calabria, Via P. Bucci, I-87036 Rende, CS, Italy; luana.malacaria@unical.it (L.M.); giuseppina.corrente@unical.it (G.A.C.); amerigo.beneduci@unical.it (A.B.); emilia.furia@unical.it (E.F.); tiziana.marino65@unical.it (T.M.)

**Keywords:** natural antioxidants, Al(III) complexes, Fe(III) complexes, chelating ability, spectroscopic characterization, DFT

## Abstract

This review focuses on the ability of some natural antioxidant molecules (i.e., hydroxycinnamic acids, coumarin-3-carboxylic acid, quercetin, luteolin and curcumin) to form Al(III)- and Fe(III)-complexes with the aim of evaluating the coordination properties from a combined experimental and theoretical point of view. Despite the contributions of previous studies on the chemical properties and biological activity of these metal complexes involving such natural antioxidants, further detailed relationships between the structure and properties are still required. In this context, the investigation on the coordination properties of Al(III) and Fe(III) toward these natural antioxidant molecules might deserve high interest to design water soluble molecule-based metal carriers that can improve the metal’s intake and/or its removal in living organisms.

## 1. Introduction

### 1.1. Role of Al(III) and Fe(III) Ions

Diseases related to the accumulation of metals, which play several critical roles in the human body, are attracting increasing attention in the scientific community [1]. Under normal conditions, some metals are considered micronutrients, being cofactors of numerous enzymes involved in many biological processes. However, they become toxic if present in relatively high amounts above a certain threshold, especially the first-row transition metal ions (e.g., Fe, Cu, and Zn), which seems to be related to severe degenerative diseases such as Alzheimer’s disease [2].

Human exposure to metals has increased over time due to their increased use, particularly with industrialization [3,4]. Toxicity due to metal exposure can also arise from their accidental ingestion through food and beverages (drinking water) [5]. Indeed, metals can be present naturally in many foods, but their uptake can be increased due to food contamination arising from some methods used in the production stages. Prevention and treatment of diseases caused by metals often occurs with the use of coordination compounds [6]. Many organic molecules, in fact, behave as ligands and can form stable complexes with metal ions, used as therapeutic agents for the treatment of metal accumulation. In particular, some transition metal complexes have shown high therapeutic potential in the treatment of different disorders, being used as antitumor, anti-inflammatory, and antidiabetic agents as well as in the treatment of diseases of the nervous system [1,3,6].

In this context, the research into suitable chelating agents against toxic metal ions overload in human tissues and the understanding of their behavior become crucial for the improvement of chelation therapy [1]. This is a promising strategy for reducing the redox stress lethal for neurons aimed at removing toxic metal ions from the human body or reducing their toxicity by transforming them into less toxic compounds or by dislocating them from the site where they exert a harmful action [6].

This review focuses on the coordination properties of aluminum(III) and iron(III) metal ions, potentially implicated in degenerative processes, since they can be targeted by metal chelators to regenerate the normal trafficking of metal [3].

The source of aluminum in humans is essentially oral intake from food, drinking water, and Al-containing drugs [1,7]. Since the 1970s, this metal has been recognized as the cause of different diseases and as a neurotoxic agent associated with encephalopathies due to its accumulation in brain tissues [8]. Currently, however, aluminum’s role in Alzheimer neuropathology is still not clear, although several experimental and clinical evidences have pointed out its implication as a primary etiological factor of Alzheimer’s disease [8,9,10].

Aluminum, with a crystal radius of 0.675 Å (coordination number VI) [11], is a typical hard metal ion. In biological systems, the majority of binding sites for it are negatively charged oxygen donors as carboxylates, phenolates, catecholates, and phosphates. The hydrolytic chemistry of aluminum affects its solubility and its bioavailability in biological environments. At pH ≤ 5, the main species is the [Al(H_2_O)_6_]^3+^ ion; the mononuclear species Al(OH)^2+^ and Al(OH)_2_^+^ are formed by the deprotonation of coordinated water molecules with increasing pH values. Polynuclear species Al_2_(OH)_2_^4+^ and Al_3_(OH)_4_^5+^ are also formed, but their concentration is strictly related to that of the total aluminum. At neutral pH, a precipitate of Al(OH)_3_ is formed, while at higher pH, the species Al(OH)_4_^−^ becomes soluble. The speciation equilibria of the soluble and insoluble forms of the hydroxo complexes and of the complexes with other competing ligands must be considered to better understand and describe the solution chemistry of aluminum in biological systems.

Due to the similar ionic radius of Al(III) and Fe(III) (0.675 Å for Al(III), 0.690 and 0.785 Å for low and high spin Fe(III), respectively) [11], Al(III) can compete with Fe(III) for binding to the biological transporter systems as transferrin. Indeed, high-spin Fe(III) is classified as a hard Lewis acid and, in analogy with Al(III), forms very stable bonds with hard ligands. Iron overload is the most common metal toxicity disease worldwide. Under normal conditions, body iron levels are governed by homeostatic controls of iron uptake, distribution, and storage. Several factors may affect iron absorption: the amount of iron present in the diet, its chemical species, the ligands coordinating iron in the intestine and the presence of other potential iron chelators. Pathological conditions may result from gastrointestinal iron absorption and/or multiple red blood cell transfusions [6].

**Scheme 1:**
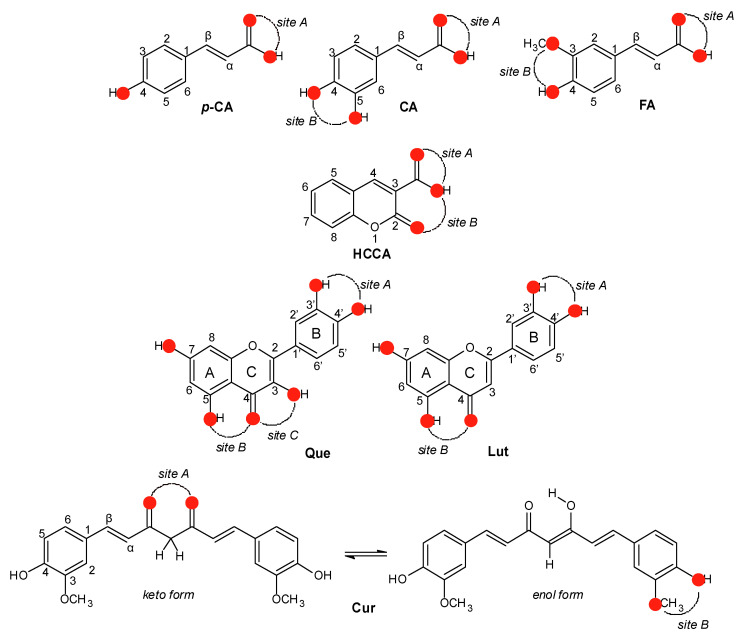
NAMs considered here for coordination of Al(III) and Fe(III) ions: *p*-coumaric (*p*-CA), caffeic (CA), ferulic (FA), and coumarin-3-carboxylic (HCCA) acids, quercetin (Que), luteolin (Lut), and curcumin (Cur). All the potential binding sites are highlighted with red circles and dashed arcs.

In this review, experimental and computational results on the ability of some natural antioxidant molecules (NAMs) (i.e., hydroxycinnamic acids, coumarin-3-carboxylic acid, quercetin, luteolin, and curcumin, Scheme 1) to form Al(III)- and Fe(III)-complexes are reported and discussed. Since the coordination chemistry background is necessary to drive the selection of drug candidates suitable to discriminate between different metal ions, the coordination properties of Al(III) and Fe(III) in aqueous solution toward the selected NAMs shown in Scheme 1 were investigated. Understanding the interactions of these metals with this class of ligands is helpful to design water soluble molecule-based metal carriers aimed to improve the metal’s intake and/or its removal in living organisms.

### 1.2. Choice of the Ligands

On the basis of the structure–activity relationship, several NAMs are well known to be effective metal chelators due to the presence of carbonyl groups as well as of phenolic hydroxyls, and carboxylic groups that, regardless of their p*K*_a_ values, can be deprotonated upon metal interaction. Thus, their ability to bind transition metal ions make these classes of molecules particularly interesting to counteract the metal-related damage. Among the numerous NAMs able to exert such activity, we focused on those derivatives whose chelating ability was investigated in aqueous solution, an environment that better mimics the physiological one.

#### 1.2.1. Hydroxycinnamic Acid Derivatives

Hydroxycinnamic acids (Scheme 1) and their derivatives are derived from cinnamic acid, an unsaturated carboxylic acid. They display interesting health benefits such as antioxidant, anti-collagenase, anti-inflammatory, antimicrobial, and anti-tyrosinase activities as well as ultraviolet (UV) protective effects [12]. The pharmacological potential exhibited by these phenolic acids and derivatives has been attributed to the presence of multiple hydroxyl groups in their chemical structure, which make them active as free radical scavengers. The presence of a double bond in the side chain leads to the possible existence of two isomeric forms: *cis* (*Z*) and *trans* (*E*). The diversity and their resultant nutraceutical properties are derived from the nature of the bonds and that of the molecules involved [13]. They are found both covalently linked to the plant cell wall polysaccharides and in their free soluble forms in the cytoplasm and are synthesized from either l-phenylalanine or l-tyrosine as part of the lignin precursor pathway.

In the present work, we focused on the sequestering ability of *p*-coumaric, caffeic, and ferulic acids (*p*-CA, CA, and FA, respectively, Scheme 1).

In *p*-CA, position 4 of the phenyl ring of the skeleton of cinnamic acid bears a hydroxyl group. It is the major precursor in the synthesis of other phenolic acids such as caffeic, chlorogenic, rosmarinic, and ferulic acids. It is widely present in fruits, vegetables, cereals, and mushrooms. Studies on *p*-CA and its conjugated forms have even revealed, besides the properties listed above, antiplatelet aggregation activity.

CA is a cinnamic acid where the phenyl ring is substituted by hydroxyl groups at positions 3 and 4. It is one of the most common phenolic acids found in fruits, vegetables, mushrooms, and herbs. It is biosynthesized by the hydroxylation of *p*-coumaric acid and has several medicinal properties, among which antidiabetic activity.

FA, known also as 4-hydroxy-3-methoxycinnamic acid, is widely distributed in beverages (coffee, beer), fruits (cabbage, potatoes, carrots), vegetables (broccoli, spinach, tomato), cereals (wheat, corn, maize), flowers, and nuts. It is a caffeic acid derivative formed by the action of the enzyme caffeate *O*-methyltransferase.

#### 1.2.2. Coumarin Derivatives

Coumarins, a family of 1,2-benzopyrones widely diffused in plants, and their derivatives have stimulated interesting research in biology and medicine due to their antioxidant, antibiotic, anticoagulant, anticancer, and anti-inflammatory properties. Coumarins can exist in a variety of forms due to the various substitutions possible in their basic structure, which modulate their biological activity. Therefore, their physicochemical properties and therapeutic applications may depend upon the substitution pattern. In addition, coumarin derivatives can yield a wide variety of metal complexes, and their complexation with d-block metal cations has been recognized as a promising route toward the development of new therapeutic agents and can be used, in principle, in specific chelation therapies [14]. Belonging to this category, the coumarin-3-carboxylic acid (HCCA) here considered, has an oxygen atom in the third position of the basic coumarin skeleton (see Scheme 1), creating a single charged ligand that can chelate, through two O donors, metal ions. HCCA behaves as a scavenger of the hydroxyl radical generated chemically or by gamma radiation, as a selective inhibitor of monoamine oxidase or as an antibacterial agent.

#### 1.2.3. Flavonoids

Flavonoids are secondary plant metabolites of the polyphenol family and form one of the most numerous and widespread families of natural substances accumulated in significant amounts in fruits and vegetables [15,16]. The increasing interest in flavonoids is due to their wide biological and pharmacological effects such as anti-cancer, anti-ulcer, anti-allergic, antioxidant, antiviral, and anti-inflammatory properties [17,18]. The anti-radical activity of flavonoids depends on their structure, the position of OH groups, and interaction with biological membranes.

Flavonoids are classified into six main subgroups according to their structure: flavan-3-ols, anthocyanins, flavonols, flavones, flavanones, and isoflavones. Many health benefits are connected with the high consumption of flavonoid-rich foods including reduced risk for heart diseases, cancer, neurodegenerative psychic diseases, and many other chronic diseases. It is assumed that oxidative stress plays an important role in the genesis of these diseases, and therefore flavones may possess therapeutic effects through antioxidant mechanisms. In addition to their antioxidant properties, flavonoids exhibit other multiple biological effects (i.e., antibacterial, antiviral, anti-inflammatory, anticarcinogenic, anti-ischemic, hypolipidemic, antimutagenic, and many others [19]. The present article reviews the coordination properties of two specific flavonoids, namely quercetin and luteolin.

Quercetin (Que) is the most commonly studied flavonol, since it can chelate the metals as a bidentate O,O-coordinating ligand. Furthermore, it is the most important and naturally occurring cancer-preventing agent [20]. The cancer preventive and therapeutic effects of quercetin have been demonstrated through in vitro as well as in vivo experimental findings [21,22]. As depicted in Scheme 1, Que consists of three phenolic rings (A, B, and C rings); it has three available sites for metal chelation including ortho-dihydroxyl (catechol) group of the B ring (site A), 5-hydroxy-4-keto group (site B), and 3-hydroxy-4-keto group (site C), since both hydroxyl and keto groups of Que have the ability to form metal complexes [23,24,25,26,27,28,29,30,31,32,33,34,35]. Interestingly, it was observed that some biological properties of quercetin such as antioxidant, antibacterial, and antitumor activities, change with metal chelation.

Luteolin (Lut) is a common flavone with several biological effects [36,37] that is often found in leaves, rinds, barks, and flowering plants. It possesses two metal ion chelating sites: the 3′,4′-dihydroxy group in ring B and the 5-hydroxy and 4-carbonyl group in ring C (see Scheme 1).

Because of poor water solubility and the bioavailability of flavonoids, their use in the food industry or pharmacy is limited. However, the formation of metal complexes may increase solubility, hydrophilicity, and bioavailability of flavonoids and therefore enlarge the area of new applications.

#### 1.2.4. Curcumin

Curcumin (1,7-bis-(4-hydroxy-3-methoxyphenyl)-1,6-heptadiene-3,5-dione, Cur) is a component of the Indian spice turmeric, manufactured from the rhizome of the perennial herb *Curcuma longa* that is widely cultivated in tropical countries in South and South East Asia, especially in China and India [38]. Curcumin is a typical example of polyphenol-rich natural remedies, and has been used for centuries in Indian traditional medicine (Ayurveda) and traditional Chinese medicine. The medicinal activity of curcumin has been known since ancient times. It has also been used as a photodynamic agent for the destruction of bacteria and tumor cells. Multiple therapeutic activities have been attributed to curcumin mostly because of its anti-inflammatory and antioxidant effects. As such, curcumin was predominantly used to treat inflammatory conditions including bronchitis, colds, parasitic worms, leprosy, arthritis, and inflammations of bladder, liver, kidney, and skin, and to improve symptoms such as fever and diarrhea. In addition, curcumin is thought to have beneficial effects in diseases of the neurological system including Alzheimer’s disease [39]. As can be evinced from Scheme 1, Cur has three chemical moieties in its structure: two aromatic ring systems containing methoxy and phenolic groups connected by a seven carbon linker consisting of an α,β-unsaturated β-diketone moiety. A keto-enol tautomeric equilibrium (as shown also in Scheme 1) characterizes the diketo moiety, which can therefore exist in different types of tautomers, depending on the environment. In the crystal state, it exists in a *cis*-enol configuration, where it is stabilized by resonance assisted hydrogen bonding and the structure consists of three substituted planar groups interconnected through two double bonds. In most of the non-polar and moderately polar solvents, the enol form is generally more stabilized than the keto form by 5 to 8 kcal mol^−1^, depending on the nature of the solvent. Due to extended conjugation, the π electron cloud is distributed along the whole molecule. In solution, it exists as *cis-trans* isomers; the *trans*-form, in which the two phenolic-methoxy groups are on the opposite sides of the curcumin backbone, is slightly more stabilized than the *cis*-form, where the phenolic methoxy groups are on the same side up the backbone [40]. The bis-keto form predominates in acidic and neutral aqueous solutions. At pH 3–7, curcumin is an extraordinarily potent H-atom donor. In the keto form, the heptadienone linkage between the two methoxyphenol rings contains a highly activated carbon atom, whose C–H bonds are very weak due to delocalization of the unpaired electron on the adjacent oxygen. In contrast, above pH 8, the enolate form of the heptadienone chain predominates, and curcumin behaves mainly as an electron donor, a more typical mechanism for phenolic antioxidant scavenging activity [41,42].

The ability of curcumin in free radical reactions may be due to the presence of two phenolic groups, the enol form of the diketone moiety, and the extended conjugated structure. Barik et al. showed that the antioxidant activity of curcumin in the β-keto-enol form is higher than those in the β-diketone form [43]. There are three factors that influence its antioxidant activity: (i) the redox state of the biological environment, (ii) the presence of metal ions, and (iii) of substituents on the side chain [44]. The strong chelating ability of diketones has been widely investigated toward some metal ions, depending on the molar ratio between metal ions and curcumin, the solvent, the metal salt, and the pH media. Over the past few decades, several studies have been published dealing with metal curcumin complexes and their applications [40,45].

## 2. Measurement of the Stability of Metal–Ligand Complexes in Aqueous Solution

The stability of metal–ligand complexes can be primarily described with three different amounts: cumulative, stepwise, and conditional stability constants. Cumulative, also called overall stability constants, are commonly indicated as β, which characterizes each complex formed in solution. If M is the metal ion, H the proton, and L the ligand, and M*_m_*H*_h_*L*_l_* is the complex formed according to the general equilibrium in Equation (1),

*m* M + *h* H + *l* L ⇆ M*_m_*H*_h_*L*_l_* (1)

β is defined as:

β = [M*_m_*H*_h_*L*_l_*]/[M]*^m^*[H]*^h^*[L]*^l^* (2)

where square brackets denote the concentrations at equilibrium. The use of concentration amounts as a replacement for the activities is usually allowed by maintaining a constant ionic strength during the experimental measurements. The overall constants are usually given as log β, and their knowledge is required for performing metal–ligand speciation calculations. In order to obtain accurate results, many experimental details have to be considered so the experimental determination of β values is complicated. β values are affected by the acid–base properties of the metal ion and ligand, by the total metal (*C*_M_) and ligand (*C*_L_) concentrations and by the pH. For this reason, the overall constants do not allow us to state the effective complex stability. Moreover, log β values cannot be used to compare the stability of complexes formed by different metal ions and ligands.

Stepwise stability constants are generally indicated as K and are commonly employed when the complexes existing in solution contain one metal ion and one or more ligands (ML*_l_*, with *l* ≥ 1). Stepwise and overall constants are related to each other: for the complexes ML and ML_2_, log β_ML_ = log *K*_ML_ and log β_ML__2_ = log *K*_ML_ + log *K*_ML__2_, respectively.

Conditional stability constants may be cumulative or stepwise and are commonly indicated with the apostrophe (i.e., β′ or *K*′). These constants can be used to compare the stability of complexes formed by different metal ions and ligands. However, the comparison is possible only when the complexes have the same stoichiometry.

The acidity constants of each ligand, the stability constants of the metal ion hydrolysis products, and the ionic product of water have to be considered to complete the speciation picture and allow speciation calculations.

For some metal–ligand complexes, more than one speciation profile has been reported in the literature, and different log β values have also been proposed. However, speciation data obtained at ionic strength as close as possible to 0.15 M and at 37 °C, which would better resemble physiological conditions, are uncommon. The information arising from these studies can be useful to identify potential complexes that can be formed in vivo; furthermore, the overall charge of the complexes is crucial in determining their partitioning in a biological environment: generally, a charged complex is hydrophilic, preferring to be solubilized in aqueous solutions (i.e., in the blood), and is unable to pass cellular barriers. In contrast, a neutral species should behave in the opposite way. Therefore, the structure of any complex, deductible from its stoichiometry, has a central role in determining its properties and toxicity.

Taking into account the importance of the metal cations and the ligands as the object of this review, results obtained from measurements carried out under physiological conditions of ionic strength and temperature (i.e., close to 0.15 M and at 37 °C) as well as the most studied temperature of 25 °C have been considered.

Table 1 reports the selected speciation information, determined by potentiometry and UV–Vis spectroscopy regarding Al(III) and Fe(III) with some NAMs, according to the general equilibrium reported in Equation (3):

*p*M^3+^ + *q*(OH)^−^ + *r*L^−^ ⇄ M*_p_*(OH)*_q_*(L)*r*^(3*p*−*q*−*r*)^     β*_pqr_* (3)

This equation takes into account the possible formation of simple (*q* = 0), mixed (*q* ≠ 0), mononuclear (*p* = 1), and polynuclear (*p* > 1) species.

The speciation profiles represent the starting point necessary for understanding the structure of metal–ligand complexes, which can be obtained from an experimental as well as theoretical approach using different characterization techniques and computational methods that will be discussed in the following sections.

## 3. Binding Sites and Complexes Formation

The ability of chelating agents to block metal-related damage has been widely explored over the past ten years and even earlier by applying quantum mechanics-based approaches [13,14,18,31,34,45,46,47,48,49,50,51,52,53]. In particular, DFT and its time dependent (TD) extension have been used to identify binding sites and complex stoichiometry, structurally characterizing the ligand–metal complexes, and comparing the outcomes with the available experimental data.

All the ligands considered in this review present more than one possible mono coordinating or chelating site, as highlighted in Scheme 1 by red circles and dashed arcs. Furthermore, to identify the most probable complexes in an aqueous environment, neutral and anionic species were taken into consideration as plausible ligands depending on the experiments or simulation conditions for complex formation [47,50,51]. From a modeling point of view, despite the wide literature on the complex formation of different antioxidants with several metal ions [18,52,53], only a few of these take into consideration the aqueous physiological environment and thus consider the metal ion surrounded by solvent molecules [13,14,31,34,45,47,51]. Some of these computational studies are supported by experiments on the complexes’ stability [13,14,31,34,45], while others have taken into account only the general physiological conditions considering neutral or deprotonated antioxidant species as a function of their p*K*_a_s [47,50,51].

**Figure 1:**
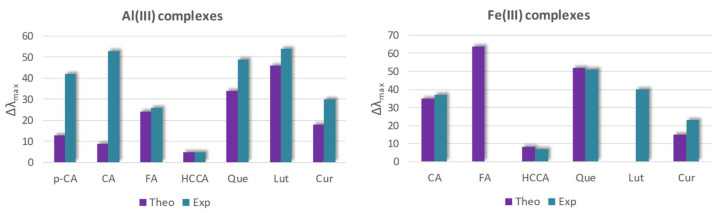
Bathochromic shift of the first absorption band upon complexation with Al(III) and Fe(III) metal ions of the different NAMs. Data were taken from references collected in Table 2, with the exception of: Fe(III)-CA spectrum recorded in aqueous solution at pH = 9 [54] and here calculated; Fe(III)-FA spectrum here calculated; Lut-Al(III) in MeOH-water (1/2 *v*/*v*) [55]; Lut-Fe(III) in EtOH [56].

In the search for the most probable binding site, key outcomes can come from spectroscopic characterization that is often used to detect the most probable complexes. Figure 1 shows the comparison of the absorption shift in the UV–Vis region of the most sensible band upon complex formation as revealed by experimental evidence and simulated by TDDFT calculations, coming from those works that better fit the aim of the present review (i.e., to describe the coordination properties of Al(III) and Fe(III) toward NAMs in aqueous environment) [13,14,31,34,45].

With the final aim of comparing the ability of the different ligands to bind Al(III) and Fe(III) metal ions in aqueous solution, metal binding affinity was here estimated (when possible on the basis of data available in the literature) by calculating the reaction free energies of the complexation reaction for the substitution of water molecules in the hexaaquo complex and considering the overall process in solution (Equation (4)):

{*n*L}*^c^* + [M(H_2_O)_6_]*^z^* →[ML*_n_*]*^z^*^+*c*^ + (6-*n-t*)H_2_O (4)

where *c* is the total formal charge of the *n* ligands; L = ligand (neutral or deprotonated) OH^−^; *z* is the charge of the hexaaquo complex; and *t* = 0, 1 takes into account the possibility that the ligands are mono or bidentate. Therefore, the formation energies of these complexes were calculated as:

(5) $$\Delta G_{f}=\Delta G({\left[ML_{n}\right]}^{z+c})+(6-nt)\Delta G(H_{2}O)-{\left(\sum_{i}^{n}\left(L\right)\right)}^{c}-\Delta G({\left[M(H_{2}O{)}_{6}\right]}^{z})$$

Most of the literature works considered here refer to measurements in aqueous solutions with pH values within the range 3.5–5, thus the accessible species and the corresponding most probable structures are of different types, depending on both the metal and ligand (Table 1) [13,14,31,34,45].

**Scheme 2:**
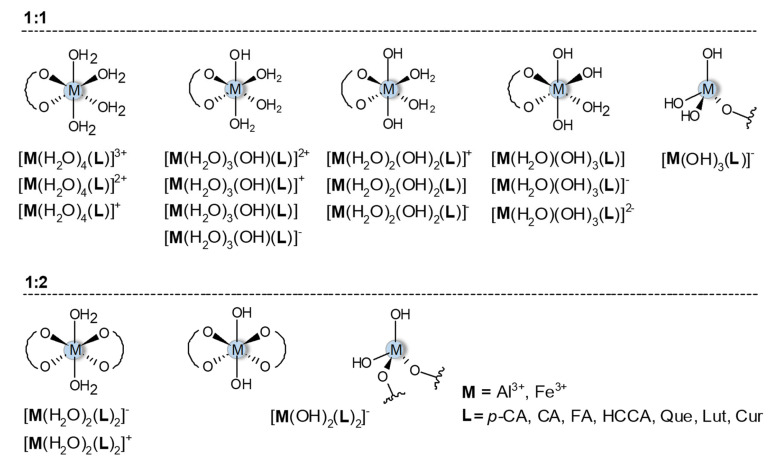
Metal–ligand complexes in aqueous solution for 1:1 and 1:2 stoichiometric ratios.

Data collected in Table 2 from the most recent studies on the selected NAMs, support the ability of Al(III) and Fe(III) metal ions to bind oxygen sites, essentially arranging in octahedral complexes (see Scheme 2). However, when metal ions and ligands exist in 1:2 stoichiometric ratios, [M(OH)_2_(L)_2_]^−^, or the ligand is monocoordinated, [M(OH)_3_L]^−^, the tetrahedral structure is plausible [14].

## 4. Complex Characterization by Spectroscopic and Spectrometric Techniques

The structural characterization of Al(III) and Fe(III) complexes with the NAMs reported in Scheme 1, is of primary importance for understanding their potential applicability. The NAM derivatives generally form complexes with a 1:1 and 1:2 metal to ligand ratio. However, the different number of binding sites offered by each of them (Scheme 1), which is very intriguing, makes a straightforward identification of the structure of the complexes more difficult. To this aim, several spectroscopic and spectrometric techniques have been used for metal-NAM complex characterization, which allows for attribution and discrimination among the different chelation sites.

Among the spectroscopic techniques, the most useful and widespread are ultraviolet-visible (UV–Vis), Fourier transform infrared (FTIR), nuclear magnetic resonance (NMR), and fluorescence spectroscopy. Mass spectrometry (MS) is also used in several instances because it provides crucial insights into the stoichiometry of the complexes. The UV–Vis characterization gives information on the d–d* transitions of the metal and on the possible geometry of the coordination complex. FTIR provides information on the functional groups of the ligand that may be involved in the complexation. The complexation is usually accompanied by a loss of some OH protons or it may cause significant proton and carbon resonance variation of the ligand close to the metal binding sites. In this context, ^1^H and ^13^C-NMR are powerful tools for elucidating the interaction between metal cations and ligands. The fluorescence emission spectra provide useful insights to identify the coordination site from the enhancement or quenching of the fluorescence as well as from the shift of the excitation and emission wavelength. Table 3 reports the results concerning the metal-to-ligand stoichiometric ratio, the solvent used for the synthesis and characterization of the complex, and the main spectroscopic and spectrometric methods used for their characterization.

In the following section, the importance of the aforementioned characterization techniques in discriminating the coordination site/sites will be highlighted, discussing the interaction of Al(III) and Fe(III) cations with hydroxycinnamic acids, coumarinic acid, quercetin, luteolin, and curcumin.

## 5. Discussion

The combination of different approaches permitted to unravel the different affinity of the two metal cations for a specific binding site of the selected NAMs. Indeed, while speciation studies allowed to predict which metal and ligand species exist in solution at a given pH, metal, and ligand total concentrations, spectroscopic, spectrometric, and computational studies provide pivotal information for identifying the preferred binding site of a metal for a specific ligand and thus the most probable complexes. All the experimental methods addressed here to describe the interactions of Al(III) and Fe(III) with the selected NAMs and information stemming from them are summarized in Table 4. 

### 5.1. Hydroxycinnamic Acid Derivatives

Al(III) ions can form 1:1 and 1:2 stoichiometric ratio species with CA and FA, and only a 1:1 stoichiometric ratio species with *p*-CA (Table 3). As can be seen in Table 1, the sequestering ability of CA toward the aluminum cation is higher than that of FA and *p*-CA as it forms complexes with four different stoichiometry. This result can be explained considering the higher solubility of CA than the other two hydroxycinnamic acids, which allows for working with a higher ligand to metal ratio, thus facilitating the water substitution in the aluminum coordination sphere [13].

**Figure 2:**
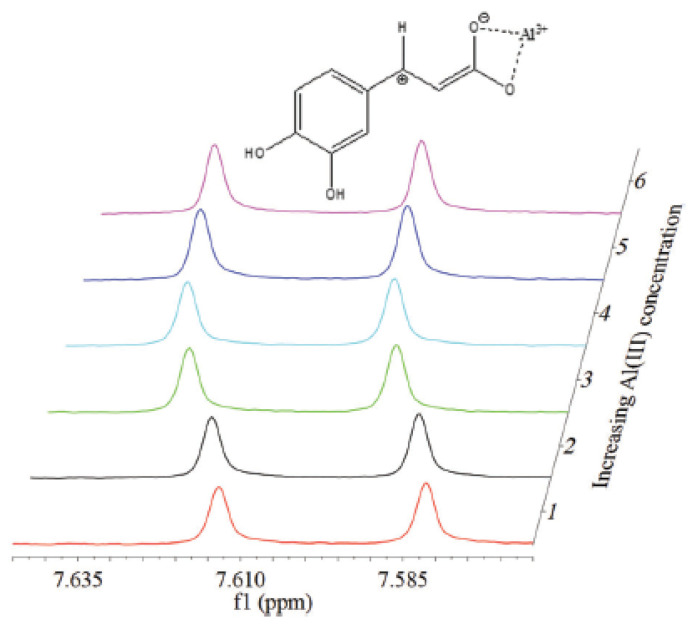
^1^H-NMR doublet of the b proton of caffeic acid at pH = 3.5 and the deshielding effect caused by the formation of the complex with Al(III). Reproduced from [14] with permission from the Centre National de la Recherche Scientifique (CNRS) and the Royal Society of Chemistry.

Metal coordination by the selected hydroxycinnamic acids can occur on the carboxylic site (site A) or on the catechol-like site (site B) in the case of CA and FA (see Scheme 1), as reported by different authors in the search for the most probable one in metal coordination [13,49,52,53]. One of the first studies in an aqueous solution (pH = 5) reported site B of CA as the preferred one for Al(III) coordination, on the basis of a comparison between recorded and simulated UV–Vis absorption spectra of selected metal–ligand complexes, identifying the three complexes of the type [M(H_2_O)_4_(L)], [M(H_2_O)_3_(OH)(L)]^−^ and [M(H_2_O)_4_(L)]^+^ (see Scheme 2) as the most plausible under such physicochemical conditions [46]. However, more recently, Beneduci et al. observed the formation of Al(III)–CA complexes by combining potentiometric measurements, UV–Vis and ^1^H-NMR spectra with computational studies. The monoanionic species were considered as the only possible CA, FA, and *p*-CA active species at pH within the range 3.5–5, thus taking into account the coordination of Al(III) only on site B of CA completely protonated, which resulted in being much more instable than the coordination on the carboxylate site, site A [13]. A similar behavior was found for FA and *p*-CA, for which the most probable complex resulted for a stoichiometric ratio (131), [Al(OH)_3_(H_2_O)(L_A_)]^−^. The formation of these complexes was clearly indicated by the huge bathochromic shift observed (up to 60 nm) in all the UV–Vis spectra with respect to those of the free ligands. According to the thermodynamic results, ligand complexation is strongly influenced by pH. In particular, it occurs just at pH 3.5 for caffeic acid, while a pH higher than 4 is needed for the other two ligands. UV–Vis spectroscopy alone cannot provide straightforward evidence on the complexation site involved. In this case, very clear insights on the structures of the complexes were obtained by ^1^H-NMR. These α, β unsaturated acids are very interesting because the doublet at about 7.6 ppm related to the proton in β can be used as an automatic alert system for the detection of changes in the electronic configuration on the carboxylic site. Thus, when the Al(III) cation interacts with this functional group, a pH-dependent shielding effect is induced, which determines a significant shift (up to 0.06 ppm) of the doublet to the lower field (Figure 2). The reason for this sensitivity is due to the positive charge delocalization on the β-carbon atom, following deprotonation caused by a pH increase or by the Al(III) complexation on the carboxylic group.

Regarding the Fe(III) complexation, no experimental structural characterization is available for CA and *p*-CA while the carboxylate anion was found as the most abundant species for FA in the complexes formed under a physiological environment in a computational work by Truong et al. They explored several complexes and concluded that the most stable ones involved the coordination of Fe(III) on site A of FA and are of the type [M(H_2_O)_4_(L)]^2+^ and [M(H_2_O)_2_(L)_2_]^+^ for 1:1 and 1:2 metal to ligand stoichiometric ratios, respectively [50]. On these complexes, we performed TDDFT calculations to get an indication of the changes in the absorption spectrum of the ligand after the complexation. Bathochromic shifts of 64 and 47 nm were found for the 1:1 and 1:2 complexes, respectively, the former are included in Figure 1. Though additional studies should be undertaken to unequivocally identify the preferred metal-to-ligand stoichiometric ratios, the most plausible complex is likely to involve the coordination of FA to Fe(III) through the carboxylate site (site A).

Interestingly, the same authors found a very similar behavior for Fe(II) metal ions, though the energies involved were of different magnitude. Data available in the literature for the Fe(II)–CA complex [47] were thus used here as a guide to calculate the ΔG_*f*_ and to simulate the absorption spectrum of a plausible Fe(III)–CA complex with the metal binding through site A. Thus, both 1:1 and 1:2 complexes with CA in its monoanionic form were considered. The simulated electronic spectra showed a red shift of 35 and 27 nm for the two complexes, respectively, and the former perfectly fits the experimental observation [54], as evident from Figure 1. Similar to the outcomes of Truong et al. [50] about FA, the obtained ΔG_*f*_ suggests a higher affinity of CA for ferrous ions rather than for the ferric one [47].

### 5.2. Coumarin Derivatives

**Figure 3:**
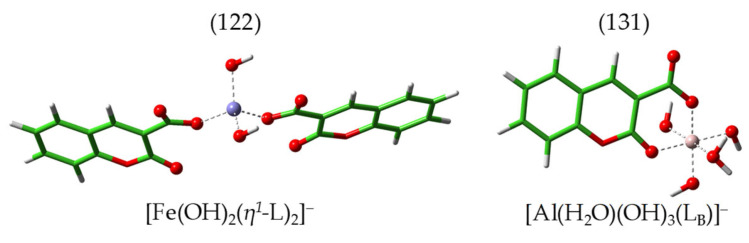
Optimized structures of the tetrahedral 1:2 Fe:HCCA and octahedral 1:1 Al:HCCA complexes.

For the aluminum–coumarin-3-carboxylic acid system, the complexes are in stoichiometric ratio 1:1 and also include hydroxyl group in the coordination sphere of the metal cation (i.e., [Al(OH)_2_(L)] and [Al(OH)_3_(L)]^−^). The stability of these species is relatively high, particularly when compared to the constant’s value for the Fe(III)–HCCA complex (see Table 1 and Table 2). The sequestering ability of HCCA toward the Al(III) cation was higher than that toward Fe(III) [14]. HCCA can form only one type of Fe(III) complex in aqueous solution (122), which contains two hydroxyl ligands together with the two anionic antioxidant molecules. In this case, two coordination modes were found as the ligand binds the metal center in η^1^ or η^2^ fashion. The former result was the most stable [Fe(OH)_2_(*η^1^*-L)_2_]^−^, in accordance with the experimental evidence (see log β in Table 1), and thus the complex arranges in a tetrahedral structure (Figure 3).

Despite a similar hard Lewis acid nature of the ions, the coordination of HCCA to the Al(III) metal ion leads to more stable complexes as evidenced by the calculated metal binding affinity (Table 2), in which the ligand chelates the metal surrounded by two or three OH^−^ ligands, [Al(H_2_O)_2_(OH)_2_(L)], and [Al(H_2_O)(OH)_3_(L)]^−^, respectively. Such structures have the metal coordination on site B, involving both the carboxylate and lactone moieties and forming a six-membered cycle. Even in the case of Al(III) complexes, the formation of a tetrahedral structure is possible, [Al(OH)_3_(L)]^−^; the ligand coordinates the metal, surrounded by three OH^−^, in η^1^ fashion. Based on the determined binding constants and the computed binding energies, the (131) stoichiometry is the most probable one at the working pH of 3.5. Furthermore, the coexistence of both tetrahedral and octahedral complexes with such a stoichiometric ratio, [Al(H_2_O)(OH)_3_(L_B_)]^−^ and [Al(OH)_3_(*η^1^*-L)]^−^, is supported by NMR measurements and by the very similar values of the calculated formation energies (Table 2). Indeed, both ^1^H- and ^13^C-NMR highlight that the overall effect caused by the complexation is a down-field shift of all signals with respect to the free ligand. It is important to note that the proton adjacent to the carboxylic acid (H in C4), is the least shifted in the ^1^H-NMR spectrum of the complex, whereas the hypothetic complexation on that moiety would have led to the opposite result. In addition, the ^13^C-NMR highlighted that the carbon atom in C2 was the most shifted, pointing out a strong interaction of the Al(III) cation with the oxygen atom of the aromatic ring. These evidences indicate that the coordination occurs through site B, which involves a relatively low electronic rearrangement of the ligand upon complex formation. Actually, the optical absorption spectrum of the free ligand was not significantly affected by the metal binding, with only a slight red shift for the Al(III)–HCCA system (see Figure 1). The excellent agreement between experiments and simulations supported the implication of site B in HCCA coordination. Overall, the HCCA prefers to bind both the metals through site B in *η^1^* and *η^2^* for Fe(III) and Al(III) ions, respectively, with a major affinity for Al(III) ion [14].

### 5.3. Flavonoids

Data on the complexation behavior of Que and Lut by Al(III) and Fe(III) ions come from several studies [26,33,64,65], some of which combine experimental and computational approaches [31,34]; others have reported the calculated structures of the complexes optimized under physiological conditions [51] while others have focused on their spectroscopic characterization [26,55,56].

Among the ligands described in this review, Que has the highest number of complexation sites, the 3′,4′-dihydroxyl groups (site A), the 5-hydroxychromone (site B), and the 3-hydroxychromone (site C), leading to an intricate and intriguing complexation behavior.

The structural characterization of the Al(III)–Que complexes in aqueous and in mixed hydro-alcoholic solutions is not very easy due to the poor solubility of quercetin and the formation of low-soluble complexes [57,66]. Useful insights on its complexation behavior come from several studies in methanol and ethanol. Ahmedova et al. reported on the synthesis of the Al(III)–Que complex in methanol and its characterization by elemental analyses, IR, and ^1^H-^13^C MAS NMR [57]. This study suggested the formation of a complex with a metal-to-ligand ratio of 1:2. The involvement of the carbonyl group in the complexation was demonstrated by a significant shift at lower energy (>50 cm^−1^) of its stretching vibration in the IR spectrum of the complex as well as by the disappearance of the broad resonance signal assigned to the intramolecular C5-OH/O = C4 hydrogen bond in the ^1^H MAS NMR. Further ^13^C MAS NMR data showed that the complex formation occurs by chelation involving the oxygen atom in position 5, which is downfield shifted according to the analysis of the electron distribution.

The complexation behavior of quercetin for iron(III) as well as the antioxidant and anti-diabetic activity of the Fe(III)–Que complexes were also studied in methanol [24,29]. Interestingly, Raza et al. reported on a 1:2 stoichiometric metal-to-ligand ratio, where iron chelates quercetin through site C [29]. This was confirmed by (i) a strong red shift (>65 nm) of the longer wavelength band in the electronic absorption spectrum, associated with the cinnamoyl system, (ii) the presence in the IR spectra of the Fe–O stretching vibration and a significant shift of the C = O stretching vibration, (iii) the ESI-MS experiments, which showed a peak at *m*/*z* 658.54 assigned to the [Fe(L)_2_]⁺ species, and (iv) the disappearance of the hydroxyl proton (3-OH) signal in the ^1^H-NMR of the complex.

Quercetin can preferentially bind Al(III) by its chelating sites B and C, as has been previously highlighted by Furia et al., who characterized a neutral 1:1 Al(III):Que complex formed in ethanol/water mixture [31]. The computational study showed that this complex, with the formula [Al(H_2_O)_2_(OH)_2_(Que)], had the lowest energy when 5-hydroxychromone was involved in the complexation, though the complex involving the coordination of the 3-hydroxychromone site was only slightly less stable. UV–Vis spectroscopy investigation showed a huge bathochromic shift of the quercetin absorption spectrum, especially of band I (429 nm vs. 368 nm), which was attributed to the conjugation of B and C rings. Strong experimental support on the involvement of the B ring in the coordination can be obtained from the comparison between the FTIR spectra of the complex and of the free ligand. The analysis of the characteristic bands of quercetin highlights a significant reduction of the stretching vibration of the carbonyl group (ν(C = O) 1639 cm^−1^ vs. 1666 cm^−1^) and of the C–C stretching vibration of the B ring (ν(C–C) 1598 cm^−1^ vs. 1611 cm^−1^) in the spectrum of the complex, which also showed the characteristic Al–O stretching vibration band of the complex at 636 cm^−1^. However, the experimental data did not allow for a clear discern between the chelating sites B and C, indicating that both complexes could be populated, as suggested by the computational results showing the kinetically possible interconversion between the two isomers in solution [31].

More recently, a comprehensive experimental and computational study on the Al(III)–Que and Fe(III)–Que complexes was conducted, for the first time, entirely in aqueous solution [34]. The speciation studies showed the formation of several complexes in the 2–5 pH range, with the precipitation of neutral solid species at higher pHs. The sequestering ability of Que toward Fe(III) was higher than that toward Al(III). Speciation profiles from potentiometric titrations showed that complexation occurs at a 1:1 ligand-to-Al(III) ratio and at 1:1 and 2:1 ligand-to-Fe(III) ratios. The stability of all these complexes was high, particularly when the hydroxyl group was involved in the coordination [34]. Computational data showed that this stability comes from the bidentate nature of the ligand. For Al(III), the most stable complex was formed when the site B was involved, irrespective of the charge of the systems. However, the speciation study showed the formation of a complex with the ligand doubly deprotonated, of the type [Al(Que)]^+^, which must necessarily involve site A in the coordination with both the hydroxyl groups deprotonated. This complex is only slightly higher in energy than the [Al(OH)(Que)]^+^, where the Al ion bound to site B, suggesting that the complex involving the catechol moiety would be kinetically favored.

**Figure 4:**
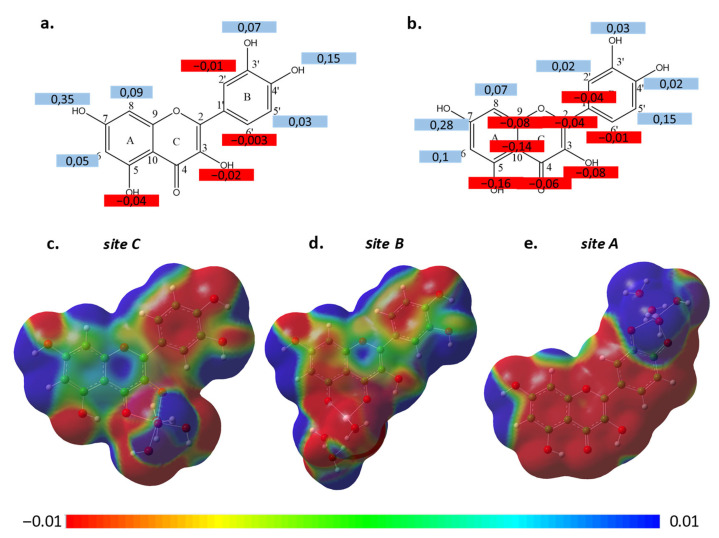
Chemical shift changes maps. (**a**) ^1^H-NMR, (**b**)^13^C-NMR. Maps of the molecular electrostatic potential (MEP) of (**c**) site C, (**d**) site B [Al(OH)_2_(Que)] complexes, and (**e**) site A [Al(OH)(Que)] complex. Reproduced from [34], Copyright Elsevier 2021.

For Fe(III), instead, among the mononuclear bidentate complexes that could be formed, the most stable was the one involving the catechol site A, with stoichiometry [Fe(OH)_2_(Que)]^−^. The neutral species formed during the titrations were characterized by ^1^H and ^13^C-NMR spectroscopy after their dissolution in DMSO. Unexpectedly, the proton spectra of the above samples showed all the OH proton signals, but with large shift (Δδ, ppm) and intensity changes compared to the free quercetin. This suggests that the dissolved solids were a mixture of different complexes that come into a rapid equilibrium in solution in the NMR time scale. It was shown that the magnitude of the chemical shift change and its sign (downfield or up-field shift) reflects a complicated electrostatic charge distribution over the entire quercetin molecule (maps of molecular electrostatic potential, MEP), which cannot be accounted for by the presence of only one complex in solution (Figure 4). Therefore, the qualitative comparison between the NMR maps of the relative chemical shift changes and the MEPs supports the hypothesis that the spectrum of the M(III)–Que mixture is a weighted average of those of the individual complexes and free quercetin. The simulated NMR spectra of the neutral complexes are consistent with the presence of a mixture of more than one complex, thus highlighting the tendency of Que to bind these metals at different coordination sites.

To our knowledge, no experimental data on the Al(III)–Lut complexes formed in aqueous solution have ever been reported. Nonetheless, a comprehensive TDDFT investigation by Amat et al. was performed on the spectroscopic properties of Al(III)–Lut complexes, where the Gibbs free energy of complex formation in water were computed considering the hexaaquo complex Al(H_2_O)_6_^3+^ as the starting reagent in the complexation reaction [51]. According to the absorption spectra obtained in methanol [55], the authors suggested the formation of three different complexes with increasing [Al^3+^]/[Lut] ratio: the 1:2 metal to ligand complex [Al(H_2_O)_2_(L_B_)_2_]^+^ and the subsequent formation of the [Al(H_2_O)_4_L_B_]^2+^ complex with a 1:1 Al:Lut stoichiometry, both with Lut chelating through site B, and the formation of two binuclear complexes with a 2:1 Al:Lut stoichiometric ratio in equilibrium between them, both involving the bidentate sites (4–5 and 3′–4′).

Looking at the ΔG_*f*_ values reported in Table 2, we note a scarce ability of Lut to bind Al(III), especially when compared with the Que complexes obtained in the same stoichiometric ratio (101), though they are calculated in a different way (see footnote of Table 2) [34,51]. In contrast, by comparing the species with the same stoichiometry (i.e., 101) between Fe(III)–Que [53] and Fe(III)–Lut [56] (see Table 1), it was possible to observe that Lut formed a slightly more stable complex than Que. This behavior could be related to the structure of the species, with a formation of five-membered and six-membered cycles, respectively. Spectroscopic and spectrometric investigations through UV–Vis, IR, ESI-MS, ESI-TOF MS, and spectrofluorometric determination were employed to study the interaction of Lut and Fe(II), Fe(III), and Al(III) [58,59]. As already noted, Lut can chelate metals by two sites: the 3′,4′-dihydroxyl group in the B ring (site A) and the 5′-hydroxy and 4-carbonyl group in the C ring (site B). Yang at al. were able to discriminate between these sites, defining the stoichiometry of the Fe(III)–Lut complexes and the chelating sites involved, by UV–Vis, IR, and ESI-MS characterization. Lut has two absorption bands in the UV–Vis region, the first one, a π−π^∗^ transition, assigned to the B ring system (cinnamoyl system) and the other, at low wavelength, representing the A ring system (benzoyl system). The UV–Vis spectra of luteolin and different Fe(III)–Lut complexes at different molar ratio (L:M = 1:1, 1:2, 1:3) acquired in ethanol solution [56] showed that the overall effect of iron addition is a bathochromic shift of these bands, with that related to the cinnamoyl system highly marked (~40 nm), and the appearance of a new band clearly indicating the complex formation. The molar ratio plot (absorbance vs. Fe(III)–Lut mole ratio) indicates the formation of a 1:1 Fe(III)–Lut complex. Significant information was obtained in this study by comparing the IR spectra of the free ligand and that of the Fe(III)–Lut complex, where many characteristic signals of the Lut cinnamoyl moiety were shifted. Among these, the most important are those assigned to the C=C stretching vibration of the benzene ring, due to an enhancement of the conjugation after coordination (38 cm^−1^), and of the C–O vibration relative to the phenolic hydroxyl groups (~13 cm^−1^), indicating their involvement in the coordination. Moreover, of noteworthy importance was the peak at 639 cm^−1^ assigned to the stretching of the O–Fe(III) bond. The ESI-MS experiments gave direct information on the 1:1 Fe(III):Lut stoichiometry of the complex since, irrespective of the molar ratio investigated (L:M = 1:1, 1:2, 1:3), the same three major peaks were always found at *m*/*z* 402.7, 431.6, and 448.8 corresponding, respectively, to the following ions: [(Lu-2H)^2−^ + Fe^3+^ + NO^3−^ + H^+^], [(Lu-2H)^2−^ + Fe^3+^ + 2 CH_3_CH_2_OH], and [(Lu-2H)^2−^ + Fe^3+^ + NO^3−^ + CH_3_CH_2_OH + H^+^]. Thus, the above data overall indicate the formation of the 1:1 M:L complex with coordination on site A.

Very recently, a thorough study on the interaction and coordination modes between Lut and iron by using electrospray ionization time-of-flight mass spectrometry (ESI-TOF MS) was reported [37]. The reaction between Lut and iron was performed in hot water to simulate the intake of this flavonoids in the human body, which is generally found in edible plants and is taken after boiling. The supernatant obtained after the reaction was measured by direct injection in the ESI-TOF MS. The results highlight the presence of a complex (*m*/*z* 626), which was assumed to be the Fe(III)–Lut 1:2 complex [37]. Moreover, analogously to other flavonoids, luteolin can reduce Fe(III) to Fe(II) under acidic conditions, leading to the formation of Fe(II)–Lut complexes [67,68]. Specifically, the presence of a 1:2 Fe(II):Lut complex was confirmed by the ESI experiment. As further evidence, the presence of Fe(II) in the Fe(III)–Lut complex solution was detected by using the indicator 1,10-phenanthroline, which forms an intense red colored complex with the Fe(II) ion.

Different photoluminescent flavonoids such as morin, luteolin, and quercetin can be used as fluorogenic ligands for metal cation detection through a chelation mechanism [69,70,71,72]. The fluorometric method can be used for the study of the chelation mechanism, since it can reduce the interference from the matrix compared with other spectroscopic methods. Sun et al. reported on the use of Lut in the spectrofluorometric determination of aluminum, based on the complex formation. Indeed, the free ligand in ethanol solution shows only a weak fluorescence due to the quenching mechanism associated with the proton transfer from the hydroxyl to the carbonyl group of the C (pyrone) ring. The formation of the Al(III)–Lut complex upon addition of Al(III) to the solution leads to a significant increase in the fluorescence emission due to the lack of the above quenching mechanism and the increase in the rigidity of the molecules that minimizes the non-radiative dissipation processes. Moreover, the emission maximum shifted considerably (>100 nm) as Al^3+^ concentration increased (from 10^−4^ to up 10^−3^ M). UV–Vis and IR spectroscopy also gave clear indication of complexation by significant signal shifts in the complex with respect to the free ligand. ESI-MS measurements provided evidence of a singly-charged complex at a *m*/*z* 579 ([Al(III) + (luteolin−2H) + (luteolin−H) + H]^+^) ratio corresponding to a 1:2 Al:Lut molar ratio. The authors proposed a dimeric structure in which one luteolin is coordinated through site A and the other through site B.

### 5.4. Curcumin

**Figure 5:**
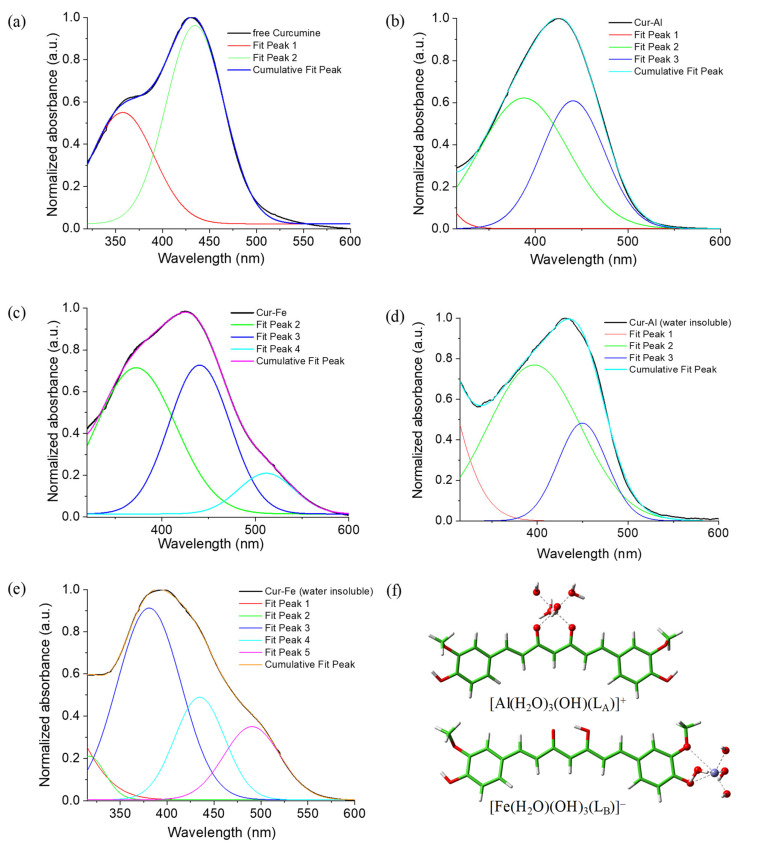
Deconvolution analysis of UV–Vis Spectra: (**a**) free Cur; (**b**) Al(III)–Cur aqueous solution; (**c**) Fe(III)–Cur aqueous solution; (**d**) Al(III)–Cur solid; (**e**) Fe(III)–Cur; and (**f**) optimized structures of the most probable Al– and Fe–Cur complexes. Adapted from [45], Copyright Elsevier 2019.

The speciation profiles obtained by potentiometric titrations in aqueous solution up to pH 4.5 showed that curcumin forms complexes with a 1:1 molar ratio, with a positive charge in the case of Al(III) and a negative one for Fe(III). At higher pH values, the formation of neutral insoluble species with a 1:2 stoichiometry occurs. The binding modes of curcumin with metal cations were evaluated combining UV–Vis and MS characterizations with computational studies [45,60]. Beneduci et al. found a different propensity of the two metal ions in the complexation with curcumin. Indeed, while Al(III) preferred the diketo moiety (by about 10 kcal mol^−1^), Fe(III) formed two almost equally stable complexes for the coordination to the keto-enolic and guaiacol sites. The UV–Vis spectrum of free curcumin showed two absorption bands, one in the UV region ascribed to the phenolic moiety and the other in the visible region (λ_max_ = 434 nm), with a shoulder at lower wavelengths. More specifically, this band reflects the equilibrium between the keto and enol forms of the curcumin and is strongly dependent on the pH value and on the type of solvent (polarity and protic/aprotic) [62,73,74]. The absorption spectra of the investigated Al–Cur complexes showed significant spectral changes with the shoulder of the Vis-band that seemed to disappear. In order to better clarify this point, the experimental Vis band of the free ligand and of the complexes were fitted with multiple Gaussian functions (Figure 5a). Indeed, this structured absorption in the free curcumin can be well deconvoluted by two bands, centered at 358 nm (assigned to the keto form) and at 434 nm (assigned to the enol form) (R^2^ > 0.999, χ^2^ < 1.3 × 10^−4^). The band deconvolution of the spectra of the complexes highlights that the enol band remained almost peaked at 434 nm, while a red shift of 30 nm was calculated for the diketo band (R^2^ > 0.998, χ^2^ < 3 × 10^−4^). This analysis revealed that the most important spectral changes could be detected in the keto-enol absorption band, thus indicating the involvement of the keto-enol site in the complexation. To support these hypotheses, a full characterization of the complexes was carried out by mass spectrometry (ESI MS/MS, LD-MS, and MS/MS). The mass spectrum, obtained by direct infusion into an electrospray mass spectrometer, clearly indicates the formation in solution of a 1:1 Al:Cur complex (signal at *m*/*z* 429), which also coordinates a water molecule and a hydroxyl moiety, as indicated by the fragmentations. The high resolution (HR) laser desorption (LD) MS and MS/MS experiments provide insight on the neutral complex obtained during titrations. Direct and consecutive fragmentations of the complex did not show specific peaks associated with the keto-enol moiety, suggesting that the coordination of the aluminum occurs via the enol oxygen with the assistance of the oxygen lone pairs of the ketonic group (Figure 5f).

The above reported spectroscopic and spectrometric characterization was also extended to investigate the ability of Fe(III) to coordinate curcumin and to study the relative coordination site. The UV–Vis spectra of the Fe(III)–Cur complexes showed significant changes in the keto-enol absorption band, which appeared much more structured than in the spectrum of free curcumin, with the appearance of a new band at around 500 nm, usually associated with a metal-to-ligand-charge transfer transition (MLCT) [62]. Gaussian deconvolution analysis showed a red shift (15 nm) of the diketo band and a relative intensity increase of this band at the expense of the enolic one with respect to the free curcumin. The increased intensity of the band assigned to the keto tautomer indicates that iron, unlike aluminum, stabilizes the keto form of curcumin. The ESI MS/MS of the neutral Fe(III)–curcumin complex (*m*/*z* 790) showed a very rich profile and many fragments could be identified to give useful insights such as those at *m*/*z* 423, probably due to the loss of a curcumin ligand, which also involves the reduction of Fe(III) to Fe(II); at *m*/*z* 177, arising from the formation of the feruloyl moiety; and at *m*/*z* 572, which is very interesting because it supports the hypothesis that the site of complexation is on the guaiacol moiety. From the LD/MS and MS/MS study, the most abundant signal resulted from the overlap of the complexes with a stoichiometry of 1:2 (M:L) containing the species [Fe(II)(Cur)_2_]^+^ (*m*/*z* 790.17) and [Fe(III)(Cur)_2_]^+^ (*m*/*z* 791.18), indicating that two molecules of curcumin chelate iron via the hydroxyl oxygen with the assistance of the oxygen lone pairs of the methoxyl groups. In addition, the formation of a fragment at *m*/*z* 599.1, due to the cleavage of the bond among the diketone functionalities, and loss of the neutral 1-aryl-3-hydroxy-1,3-butadiene moiety, confirms the results obtained by ESI MS/MS that iron coordination occurs on the guaiacol moiety (Figure 5f).

Other studies on the complexation between curcumin and iron (Fe(II) and Fe(III)) in methanol or methanol/water mixtures have been reported in the last few decades, showing the formation of complexes with a 1:2 M:L molar ratio where the ligand behaves as a bidentate by chelating the metal ion with the keto-enol moiety [61,62,63,75,76]. Generally, this coordination mode is accompanied by a hypsochromic shift in the UV–Vis spectrum of the complex with respect to the free curcumin [63] as well as by a significant shift (~40 cm^−1^) in the IR spectrum of the complex of the following vibrations: ν(CO)keto, δ(CO)enol, and ν(C=C) aromatic [62].

## 6. Conclusions

In this review, experimental and computational results on the ability of some natural antioxidant molecules, NAMs, to form Al(III)– and Fe(III)–complexes are collected. The chelating ability of selected NAMs (i.e., hydroxycinnamic acids (*p*-coumaric, caffeic and ferulic acids), coumarin-3-carboxylic acid, quercetin, luteolin and curcumin), well known to be effective metal chelators, was investigated in aqueous solution, an environment that better mimics the physiological one. Data collected here evidence that:

- hydroxycinnamic acids (*p*CA, CA, and FA) are able to form more stable complexes with Al(III) than with Fe(III) coordinating the metal ion through the carboxylate site in all cases.
- coumarin-3-carboxylic acid, similarly, prefers to bind Al(III) rather than Fe(III), forming 1:1 and 1:2 M:L stoichiometric ratio complexes, respectively. Consequently, octahedral complexes with Al(III), involving both carboxylate and lactone moieties, and tetrahedral complex with Fe(III) in a *η^1^* ligand’s coordination were analyzed.
- flavonoids (Que and Lut) formed 1:1 and 1:2 M:L complexes with both metal ions, though they showed a more intricate behavior, as more than one coordination mode was found plausible with both metal ions, making the identification of the preferred coordination site and thus the most probable complex in a water environment, especially in the case of Que, difficult.
- Curcumin discriminates well between the two metal ions since it prefers to coordinate Al(III) through the diketo site while the Fe(III) results most probably bound to the guaiacol site.

The excursus of data available for the selected NAMs toward bioavailable Al(III) and Fe(III) metal ions confirms the ability of phenolic compounds to trap metals preventing their accumulation and their harmful action to human health.

## Figures and Tables

**Table 1:** Formation of complexes of some natural antioxidants with Al(III) and Fe(III), according to Equation (3). Standard deviations are reported as 3σ.

Al(III)				
Ligand	(pqr)	log β	Temperature	Reference
CA	(111)	13.40 ± 0.03	37 °C	[13]
	(121)	22.26 ± 0.06		
	(131)	30.87 ± 0.09		
	(142)	42.53 ± 0.09		
FA	(121)	21.3 ± 0.3	37 °C	[13]
	(131)	30.31 ± 0.02		
	(142)	42.24 ± 0.02		
*p*-CA	(131)	30.21 ± 0.09	37 °C	[13]
HCCA	(121)	25.9 ± 0.2	37 °C	[14]
	(131)	36.5 ± 0.3		
Que	(121)	29.25 ± 0.06	37 °C	[34]
	(111)	16.1 ± 0.1	37 °C	[26]
	(101)	23.0 ± 0.5	25 °C	[27]
Cur	(111)	16.4 ± 0.1	37 °C	[45]
**Fe(III)**				
HCCA	(122)	28.35 ± 0.06	37 °C	[14]
Que	(121)	37.24 ± 0.06	37 °C	[26]
	(112)	43.9 ± 0.1		
	(122)	53.1 ± 0.1		
	(101)	5.5	25 °C	[46]
	(102)	9.56		
Lut	(101)	8.4	25 °C	[47]
Cur	(131)	41.4 ± 0.3	37 °C	[45]

**Table 2:** Free energy formation (ΔG*_f_*) of the complexes formed between the selected natural antioxidants and Al(III) or Fe(III), calculated according to Equation (5).

	Al(III)			
Ligand (L)	Complex	ΔG_*f*_	Level of Theory	Reference
CA	[Al(H_2_O)_3_(OH)(L_A_)]^+^	−119.4	M052X/6-31+G(d) − SMD, water	[13]
FA	[Al(H_2_O)(OH)_3_(L_A_)]^−^	−158.4	M052X/6-31+G(d) − SMD, water	[13]
*p*-CA	[Al(H_2_O)(OH)_3_(L_A_)]^−^	−159.5	M052X/6-31+G(d) − SMD, water	[13]
HCCA	[Al(H_2_O)_2_(OH)_2_(L_B_)]	−145.3	M052X/6-31+G(d) − SMD, water	[14]
	[Al(H_2_O)_2_(OH)_2_(L_A_)]	−139.5		
	[Al(H_2_O)(OH)_3_(L_B_)]^−^	−157.7		
	[Al(OH)_3_(*η^1^-*L_A_)]^−^	−159.0		
Que	[Al(H_2_O)_2_(OH)_2_(L_B_)]	−123.7	M052X/6-31+G(d) − SMD, ethanol	[31]
	[Al(H_2_O)_2_(OH)_2_(L_C_)]	−119.0		
	[Al(H_2_O)_3_(OH)(L_B_)]^+^	−71.2	M052X/6-31+G(d) − SMD, water	[34]
	[Al(H_2_O)_3_(OH)(L_C_)]^+^	−68.6		
	[Al(H_2_O)_4_(L_A_)]^+^	−55.9		
Lut	[Al(H_2_O)_4_(L_A_)]^2+^	−1.5	B3LYP/6-31G ** − CPCM, water	[51] ^a^
	[Al(H_2_O)_4_(L_B_)]^2+^	2.5		
	[Al(H_2_O)_2_(L_B_)_2_]^+^	−6.8		
	[Al(H_2_O)_2_(L_A_)_2_]^+^	−1.1		
Cur	[Al(H_2_O)_3_(OH)(L_A_)]^+^	−135.1	M052X/6-31+G(d) − SMD, water	[45]
	[Al(H_2_O)_3_(OH)(L_B_)]^+^	−124.9		
	**Fe**(**III**)			
CA	[Fe(H_2_O)_4_(L_A_)]^2+^	5.8	M052X/6-31+G(d) − SMD, water	This work
	[Fe(H_2_O)_4_(L_A_)_2_]^+^	16.0		
FA	[Fe(H_2_O)_4_(L_A_)]^2+^	–49.7	M05/6-311++G(d,p) − SMD, water	[50]
	[Fe(H_2_O)_4_(L_A_)_2_]^+^	–85.4		
HCCA	[Fe(OH)_2_(*η^1^*-L)_2_]^−^	−27.1	M052X/6-31+G(d) − SMD, water	[14]
	[Fe(OH)_2_(*η^2^*-L)_2_]^−^	−19.2		
Que	[Fe(H_2_O)_2_(OH)_2_(L_A_)]^−^	−99.4	M052X/6-31+G(d) − SMD, water	[34]
	[Fe(H_2_O)(OH)(L_A_)_2_]^−^	−113.7		
	[Fe(OH)_2_(L_A_)_2_]^−^	−125.8		
Cur	[Fe(H_2_O)(OH)_3_(L_A_)]^−^	−57.1	M052X/6-31+G(d) − SMD, water	[45]
	[Fe(H_2_O)(OH)_3_(L_B_)]^−^	−55.5		

^a^ Optimization in vacuum, single point in solvent; ΔG_f_ for the reaction nAl(H_2_O)_6_^3+^ + mL Al_n_X^y^L^k^_m_ + (l − (3n − k))H_2_O + (3n − k)H_3_O^+^, where l is the number of water molecules detached from Al(H_2_O)_6_^3+^ upon ligand complexation by using the experimental value of -ΔG_vap_(H_2_O) as ΔG_solv_(H_2_O) in the calculation of the Gibbs free energy for the formation of one mole of the considered complexes.

**Table 3:** Methods used to characterize the complexes between the selected natural antioxidants and Al(III) or Fe(III) in the reported metal to ligand (M:L) stoichiometric ratios.

	Al(III)			
Ligand	M:L	Solvent	Characterization Methods	Reference
CA	1:1 1:2	water	UV–Vis, ^1^H-NMR	[13]
FA	1:1 1:2	water	UV–Vis, ^1^H-NMR	[13]
*p*-CA	1:1	water	UV–Vis, ^1^H-NMR	[13]
HCCA	1:1	water	UV–Vis, ^1^H, ^13^C-NMR	[14]
Que	1:2	methanol	FTIR, ^1^H, ^13^C MAS NMR	[57]
	1:1	water	UV–Vis, ^1^H, ^13^C-NMR	[34]
Lut	1:1 2:1	methanol:water (9:1)	UV–Vis, FT-IR, RAMAN	[58]
	1:2	ethanol	UV–Vis, FTIR, fluorescence, ESI-MS	[59]
Cur	1:1 2:1 3:1	methanol	^1^H, ^13^C, ^27^Al-NMR, MALDI-TOF	[60]
	1:1 1:2	water	UV-Vis, ESI MS/MS, LD-MS, MS/MS	[45]
	**Fe**(**III**)			
HCCA	1:2	water	UV–Vis, ^1^H, ^13^C-NMR	[14]
Que	1:2	methanol	UV–Vis, FTIR, ESI MS, ^1^H-NMR	[29]
	1:2	water	UV–Vis, ^1^H, ^13^C-NMR	[34]
Lut	1:1	ethanol	UV–Vis, FT-IR, ESI MS	[56]
	1:2 1:2 ^a^	water	ESI MS, ESI-TOF MS	[37]
Cur	1:2	water	UV–Vis, ESI MS/MS, LD-MS, MS/MS	[45]
	1:2	methanol	FTIR	[61]
	1:2	water:methanol (1:1)	UV–Vis, FTIR, RAMAN, ESR, ^1^H-NMR, X-ray	[62]
	1:2	methanol	UV–Vis, FTIR	[63]

a. Fe(II)-Lut [37].

**Table 4:** Details of the identification and characterization methods.

Experimental Methods	Methodology	Principle	Analysis Results	Reliability and Quality of the Results on the Complex Formation
Potentiometry	✓ Measurements in aqueous solution	The complexation equilibria are studied by measuring, with a glass electrode, the competition of the ligand for H^+^ and metal cations.	The experimental data, obtained at different metal and ligand total concentrations, and processed by numerical procedures, were rationalized according to a general equilibrium, to obtain the complexes existing in solution at a given pH.	Speciation studies allow to predict the most probable stoichiometric coefficients of the complexes and the corresponding stability constants.
UV-Vis	✓ Spectroscopic technique ✓ No sample preparation ✓ Measure in solution	Absorption of ultraviolet and visible photons by a molecule causes a change from its fundamental electronic state to an excited electronic state.	In a typical UV–visible spectrum the wavelength is reported in the abscissa (190 < λ < 780 nm) and the absorbance (or the transmittance) in the ordinate. The recorded spectrum will be characterized by a series of bands of variable intensity.	Complexation leads to specific band shifts in the spectrum of the ligand that, in several cases, can be directly related to the involvement of a specific complexation site of the ligand. In addition, metal-to-ligand charge transfer transitions and d-d transition bands may occur
^1^H, ^13^C, ^27^Al-NMR	✓ Spectroscopic technique ✓ Measure in solids and in solution	Absorption of a radio frequency radiation is measured after immersing a molecule in a strong static magnetic field, which causes nuclear spin transitions.	An NMR spectrum shows the frequency absorbed and then emitted by the atoms of the nucleus under examination, which depends on the chemical environment around it (chemical shift).	Upon complexation huge shielding or deshielding effects may be induced on the magnetic nuclei of the ligand, generally placed close to the binding site. However, significant chemical shift displacement can occur far from the binding site when resonance structures are involved in the ligand. Quantitative information on the structural properties of the complexes may also be obtained from the spectra of magnetic metals such as ^27^Al.
FTIR	✓ Spectroscopic technique ✓ Measure in solution and in solid	Absorption of an infrared photon by a molecule, causes its transition from its fundamental vibrational state to an excited vibrational state.	In a typical IR spectrum, the percentage of transmittance is plotted against the wave number (4000 cm^−1^ < λ < 400 cm^−1^). Each peak in the spectrum can be assigned to a specific functional group.	Formation of a complex usually leads to the appearance of specific peaks related to the metal-heteroatom bond (e.g., Al-O). Moreover, metal binding affects the bond vibrational energies of the functional groups of the ligand involved in the complexation, leading to related peak shifts.
Mass spectrometry	✓ Spectrometric techniques ✓ Measure in solution and in solid ✓ Destructive technique	This technique allows separating a mixture of ions according to their mass/charge ratio. Molecules are ionized and fragmented into lighter ions according to typical patterns depending on their chemical structure.	The diagram showing the abundance of each ion as a function of the mass/charge ratio (*m*/*z*) is the so-called mass spectrum, typical of each compound as it is directly related to its chemical structure and to the ionization conditions it has been subjected.	Upon complexation, specific *m*/*z* fragments containing the metal can be detected, indicative of the specific binding site involved. The stoichiometry of the complex (M:L ratio) can be easily determined.
Fluorescence	✓ Spectroscopic technique ✓ Measure in solution ✓ Non-destructive technique	An incident photon excites the fluorophore from the ground state to a higher energy state (electronically and vibrationally) with the same spin. In a few nanoseconds, the excited electron returns to the ground electronic state passing through one or more excited states at intermediate energy. All decays except one are usually non-radiative, while the last one emits light with a longer wavelength than the incident radiation, that is the fluorescence.	In a typical fluorescence spectrum, the fluorescence intensity is plotted vs. the wavelength of emission.	Complex formation between a metal and a fluorophore may lead to a significant fluorescence enhancement due to restricted intramolecular rotations of the ligand, as well as a huge shift of the emission maximum.
RAMAN	✓ Spectroscopic technique ✓ Measure in solution, solid and gas phase ✓ Non-destructive technique	This technique is based on the diffusion (scattering) of a monochromatic electromagnetic radiation by the analyzed sample. The diffused radiation contains the components with different energy (Rayleigh, Stokes and anti-Stokes) associated to molecular vibrations of different functional groups.	In a Raman spectrum the intensity of the signals proportional to the number of Stokes photons, are plotted against the Raman shift (in cm^−1)^, corresponding to the energy difference associated to transitions between fundamental vibrational levels.	Upon complexation specific band shifts (i.e., inplane skeletal vibrations as well as stretching vibrations) occurs in the RAMAN spectra indicating which part of the ligand takes part in the metal binding.
ESR	✓ Spectroscopic technique ✓ Measure in solution	It detects the induced transition by a microwave radiation between the energy levels of electron spins under a static magnetic field. It allows the study of organic and inorganic free radicals, odd electrons molecules, molecules in the triplet state, transition metal complexes, etc.	The EPR spectrum is the first derivative of the absorption spectrum obtained in the microwave range	ESR measurements of the magnetic moment, is indicative of low or high spin configuration around the metal, thus providing information on the coordination geometry
X-ray Diffraction	✓ Spectroscopic technique ✓ Measure on powder ✓ Non-destructive technique	It allows the structural characterization of crystalline materials. It is based on the constructive interference of an x-ray monochromatic beam which is scattered at specific angles from each set of lattice planes in a sample.	The x-ray pattern is the fingerprint of the periodic atomic arrangement in the material	X-ray diffraction provides insight on the coordination geometry around the metal ion as well as on the degree of crystallinity of the complexes.
